# AK-I-190, a New Catalytic Inhibitor of Topoisomerase II with Anti-Proliferative and Pro-Apoptotic Activity on Androgen-Negative Prostate Cancer Cells

**DOI:** 10.3390/ijms222011246

**Published:** 2021-10-18

**Authors:** Kyung-Hwa Jeon, Seojeong Park, Hae Jin Jang, Soo-Yeon Hwang, Aarajana Shrestha, Eung-Seok Lee, Youngjoo Kwon

**Affiliations:** 1 College of Pharmacy, Graduate School of Pharmaceutical Sciences, Ewha Womans University, Seoul 03760, Korea; kjeon@ewha.ac.kr (K.-H.J.); tyfpzk@naver.com (S.P.); kate0915@hanmail.net (H.J.J.); hilarious06@naver.com (S.-Y.H.); 2 College of Pharmacy, Yeungnam University, Gyeongsan 38541, Korea; aarajana90@gmail.com (A.S.); eslee@yu.ac.kr (E.-S.L.)

**Keywords:** topoisomerase II inhibitor, CRPC, DNA intercalator

## Abstract

Castration-resistant prostate cancer (CRPC) is a clinical challenge in treatment because of its aggressive nature and resistance to androgen deprivation therapy. Topoisomerase II catalytic inhibitors have been suggested as a strategy to overcome these issues. We previously reported AK-I-190 as a novel topoisomerase II inhibitor. In this study, the mechanism of AK-I-190 was clarified using various types of spectroscopic and biological evaluations. AK-I-190 showed potent topoisomerase II inhibitory activity through intercalating into DNA without stabilizing the DNA-enzyme cleavage complex, resulting in significantly less DNA toxicity than etoposide, a clinically used topoisomerase II poison. AK-I-190 induced G1 arrest and effectively inhibited cell proliferation and colony formation in combination with paclitaxel in an androgen receptor–negative CRPC cell line. Our results confirmed that topoisomerase II catalytic inhibition inhibited proliferation and induced apoptosis of AR-independently growing prostate cancer cells. These findings indicate the clinical relevance of topoisomerase II catalytic inhibitors in androgen receptor-negative prostate cancer.

## 1. Introduction

Prostate cancer (PCa) is one of the most common types of cancer in elderly men [1]. This type of cancer is driven by the androgen receptor (AR) signaling pathway, and therefore AR-targeting drugs in androgen-deprivation therapy are currently the primary therapeutic option for PCa [2]. AR-targeting treatment results in significant reduction of tumoral growth and progression at all stages of PCa [3]. However, in a few cases, the tumor adapts to AR suppression through persistent activation of AR signaling without ligand or complete elimination in AR expression, leading to recurrent and metastatic cancer [4,5].

After exposure to androgen-deprivation therapy, AR-positive PCa cells undergo lineage plasticity and epigenetic reprograming [6]. During this process, the development of stem cell likeness transforms PCa cells to gain androgen independence in cellular growth, so-called castration resistance [7]. Castration-resistant prostate cancer (CRPC) has emerged as a major obstacle in PCa treatment. It is strongly believed that alternative oncogenic signaling pathways are activated to bypass androgen dependency, which leads to CRPC development. Therefore, to overcome castration resistance and effectively manage PCa, a therapeutic strategy involving additional targets in addition to targeting AR signaling is required. 

Topoisomerase II is an enzyme that resolves topological issues in the process of replication, transcription, recombination, chromosomal condensation and segregation [8]. Because of the highly proliferative nature of cancer cells, topoisomerase II inhibitors, such as etoposide, doxorubicin and mitoxantrone, have long been used in diverse types of solid tumors. These topoisomerase II inhibitors have also been suggested for PCa treatment. Mitoxantrone was approved by the FDA in 1996 for the treatment of PCa, and several studies have shown clinical benefits of etoposide in combination regimens for the treatment of patients with CRPC [9,10,11].

**Figure 1:**
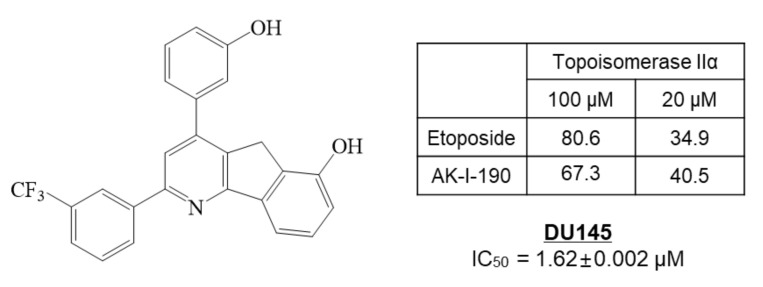
The structure and topoisomerase II inhibitory and anti-proliferative activities of AK-I-190 (2-(3-trifluorophenyl)-4-(3-hydroxyphenyl)-5*H*-indeno[1,2-*b*]pyridin-6-ol) in DU145 prostate cancer cells.

In our previous study, we synthesized 172 new halogenated 2,4-diphenyl Indeno[1,2-*b*]pyridinol derivatives as topoisomerase inhibitors [12]. Among the inhibitors, AK-I-190, 2-(3-trifluorophenyl)-4-(3-hydroxyphenyl)-5*H*-indeno[1,2-*b*]pyridin-6-ol, showed potent topoisomerase II inhibition with anti-proliferative activity in the DU145 AR-negative PCa cell line (Figure 1). In this study, we clarified the molecular mechanism of AK-I-190 as a DNA intercalating catalytic inhibitor of topoisomerase II. We further evaluated the anti-proliferative and pro-apoptotic activities of AK-I-190 in AR-independently growing PCa cells. Our results suggest the clinical relevance and efficacy of the topoisomerase II catalytic inhibitor in AR-negative PCa.

## 2. Results

### 2.1. Topoisomerase II Inhibitory Activity of AK-I-190 as a Catalytic Inhibitor

**Figure 2:**
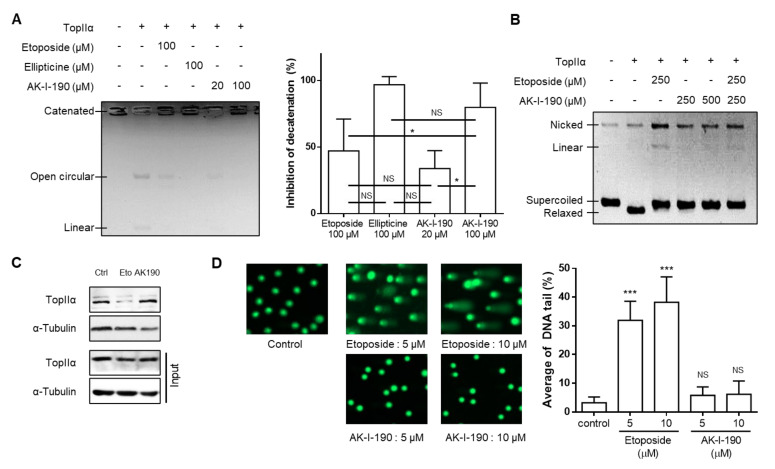
AK-I-190 inhibits topoisomerase II inhibition as a catalytic inhibitor. (**A**) kDNA decatenation assay of AK-I-190. The graph on the right shows quantification of the values measured in the gel. (**B**) Cleavage complex assay. Supercoiled DNA (pBR322) was pre-incubated with DNA topoisomerase IIα and the indicated compound was added. After incubation and agarose gel electrophoresis, bands of linear DNA were analyzed. (**C**) Band depletion assay. Formation of topoisomerase IIα–DNA cleavage complex was assessed using the band depletion assay. Cells treated with vehicle, etoposide, or AK-I-190 were lysed with denaturing buffer and centrifuged. DNA-unbound topoisomerase IIα was evaluated in the supernatant (upper panel). The whole cell lysate in the same experimental condition was used as input. (**D**) Alkaline comet assay. Comet slides were electrophoresed at high pH and stained with a SYBR gold solution. The comet tails were observed by fluorescence microscopy. Quantification of DNA tails was conducted using Komet 5.5 software. The intensities of comet tails versus comet heads reflected the extent of DNA breaks (*n* = 50). * *p* < 0.05 and *** *p* < 0.001 vs. control; NS, not significant.

We evaluated the DNA topoisomerase II inhibitory activity of AK-I-190, synthesized as previously reported [12], using the kinetoplast DNA (kDNA) decatenation assay. This experimental method is used for the assessment of topoisomerase II-specific decatenating ability [13]. DNA topoisomerase II, but not DNA topoisomerase I, has the ability to decatenate DNA due to its ability to cleave DNA double strands simultaneously, which is essential for cells that hyper-proliferate through the process of mitosis and cytokinesis [9]. Our results showed that AK-I-190 treatment retained kDNA in the catenated form (Figure 2A). Notably, the same concentration of AK-I-190 (100 μM) showed better or equivalent inhibitory activity compared with the known topoisomerase II inhibitors etoposide and ellipticine. We next evaluated whether AK-I-190 was capable of stabilizing the DNA-enzyme complex. Stabilization of the topoisomerase-DNA cleavage complex produces linear DNA, which is a cause of adverse effects induced by topoisomerase poison, including etoposide [14]. In the cleavage complex assay, the linear form of DNA was not generated by AK-I-190, whereas etoposide treatment resulted in a linear DNA band (Figure 2B). 

As our results showed that AK-I-190 effectively inhibited enzymatic activity without induction of linearized DNA, a band depletion assay was conducted to determine whether AK-I-190 was a poison or a catalytic inhibitor. Treatment with etoposide, but not AK-I-190, resulted in a significant decrease in free topoisomerase IIα level, indicating that topoisomerase IIα covalently bound to DNA was increased in etoposide-treated cells (Figure 2C). Truncated DNA induced by stabilization of the enzyme-DNA complex was confirmed by alkaline comet assay (Figure 2D). Unlike etoposide, AK-I-190 did not result in an increase in DNA tail compared with controls. Collectively, these results indicate that AK-I-190 is a catalytic inhibitor of topoisomerase II rather than a poison.

### 2.2. DNA Intercalation of AK-I-190

**Figure 3:**
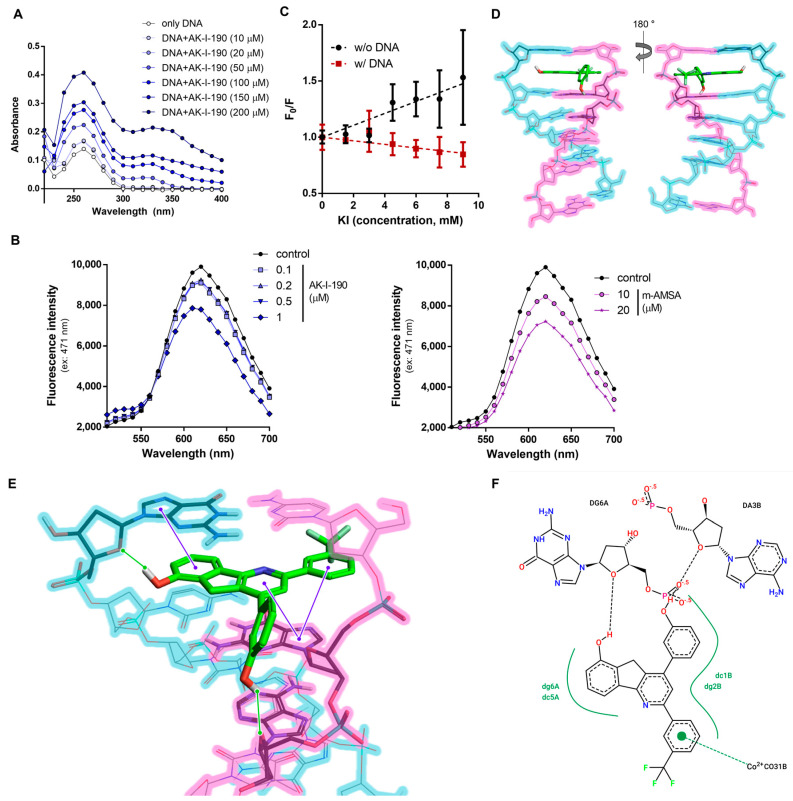
DNA intercalation of AK-I-190. (**A**) Interaction of AK-I-190 with ct-DNA was monitored using UV/vis spectroscopy. UV/vis spectrum for ct-DNA (200 μM) in the presence of various AK-I-190 concentrations (0–200 μM) in 10 mM Tris buffer (pH 7.4). (**B**) Fluorescence spectra during titration of AK-I-190 to the complex of ct-DNA with EtBr (intercalator). The EtBr-DNA complex was excited at 471 nm and emission spectra were recorded from 500 nm to 700 nm. Fluorescence intensity decreased with subsequent addition of AK-I-190 (left) and m-AMSA (right). (**C**) KI quenching experiment. Stern–Volmer plot of fluorescence quenching of AK-I-190 (100 μM) by increasing KI concentration (0–9 mM) in 10 mM Tris buffer (pH 7.4) in the presence (red) and absence (black) of ct-DNA. (**D**,**E**) Molecular docking study of AK-I-190. Docking studies of AK-I-190 were carried out using the X-ray crystal structure of human DNA complexed with ellipticine as a template (PDB code, 1Z3F) using the Flare program. AK-I-190 potentially binds to DNA double helix by intercalation between base pairs. Each DNA strand is shown as blue and pink highlighted lines. Residues participating in the interaction are indicated by bold sticks and AK-I-190 is shown by capped sticks. Both are colored according to atom (carbon, black or green; oxygen, red; nitrogen, blue; phosphorus, cyan; fluoride, light green). Expected hydrogen bonds are indicated as green solid lines and π–π interactions are represented as purple solid lines. (**F**) Two-dimensional diagram of the interaction between AK-I-190 and the DNA double helical structure. The diagram was created using Pose View in proteins.plus/ (accessed on 20 August 2021).

For a deeper understanding of the mechanism of topoisomerase II inhibitory activity, we analyzed AK-I-190 using various spectroscopic methods. There are several known modes of action of topoisomerase II–targeting drugs, including stabilizing the cleavage complex, inhibiting ATPase activity of topoisomerase II, chelating metal ions essential for topoisomerase II function, and inhibiting DNA access to topoisomerase II by binding to DNA. As our results indicate that AK-I-190 is a catalytic inhibitor of topoisomerase II, we evaluated AK-I-190 activity in each mode of action except for cleavage complex stabilization. AK-I-190 did not inhibit topoisomerase II ATPase activity and had no chelating ability [14] (Supplementary Figure S1). For the evaluation of DNA binding, the absorption spectra of DNA with diverse concentrations of AK-I-190 were analyzed (Figure 3A). Titrating AK-I-190 in DNA solution increased absorbance, confirming that AK-I-190 was bound to DNA. The absorbance values of 200 μM calf thymus DNA (ct-DNA) and 200 μM AK-I-190 at 260 nm wavelength were 0.174 and 0.075, respectively. The absorbance of a mixture of the same concentration of ct-DNA and AK-I-190 (200 μM each) increased to 0.350 at 260 nm. This result indicates that AK-I-190 interacts with DNA, thereby attenuating enzymatic activity of topoisomerase II. 

We next examined the binding mode of AK-I-190 to DNA using EtBr displacement and KI quenching assays. AK-I-190 was confirmed to interact with DNA in an intercalating manner similar to that of m-AMSA, a known DNA intercalating agent (Figure 3B,C) [15]. The fluorescence quenching ability of iodide ions on AK-I-190 was evaluated in the absence and presence of DNA co-treatment. The fluorescence of the DNA intercalating agent tends to weaken the fluorescence quenching ability of iodide ions under the DNA co-treatment condition because of the intercalating action, and thus the Fo/F ratio value tends to decrease as the KI concentration increases. The Stern–Volmer quenching constant (K_SV_) value of iodide ion was 52.99 M^−1^ in the absence of ct-DNA, whereas its fluorescence-quenching ability was completely diminished by ct-DNA addition. AK-I-190 was revealed to be a DNA intercalating agent and not a groove binder. 

DNA intercalation of AK-I-190 was further analyzed using a docking study (Figure 3D–F). The results revealed that two hydroxyl groups of AK-I-190 formed hydrogen bonds with each chain of DNA, and the ring structure participated in multiple π–π interactions with purine and pyrimidine rings of DNA. The flat structure of AK-I-190 was responsible for the DNA intercalation and the additional hydrogen bonds were for anchorage.

### 2.3. G1 Arrest and Apoptotic Induction of AK-I-190

**Figure 4:**
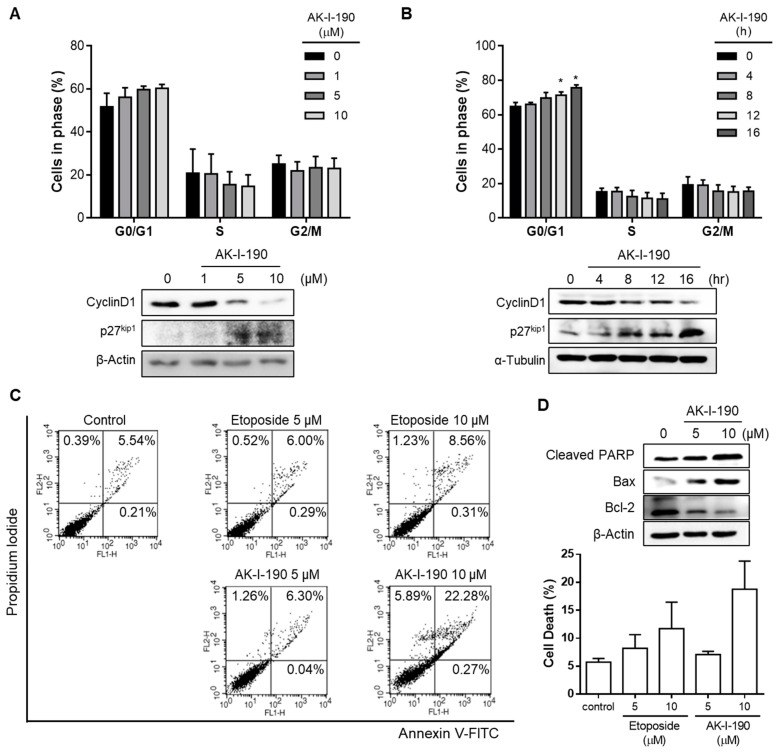
Apoptosis induced by AK-I-190. (**A**,**B**) Cell cycle analysis of AK-I-190-treated DU145 cells. G1 arrest was observed in AK-I-190 concentration- (**A**) and time-dependent (**B**) manners. * *p* < 0.05 vs. Western blot analysis of G1 checkpoint markers, cyclin D1 and p27^kip1^, in DU145 cells after treatment of AK-I-190. (**C**) Apoptosis induction assessed using Annexin V–PI double staining. DU145 cells were treated with varying concentrations of AK-I-191 for 36 h. Apoptotic cells were detected by flow cytometry and plotted as Annexin V-positive cells on the right side. (**D**) Evaluation of apoptosis at the protein expression level. Protein levels of pro- and anti-apoptotic proteins bcl-2, bax, and cleaved PARP were analyzed using Western blotting.

We next evaluated the effect of the topoisomerase II inhibition activity by AK-I-190 on PCa cells by cell cycle analysis (Figure 4A,B). Flow cytometric analysis revealed that AK-I-190 effectively induced a G1 arrest in DU145 cells in a time- and concentration-dependent manner. Western blot analysis further confirmed that AK-I-190 downregulated the expression level of cyclin D1, a protein related to G1/S progression, and elevated the expression level of p27^kip1^, a CDK-related inhibitor, in a time- and concentration-dependent manner. These results indicate that AK-I-190 successfully induced cell cycle arrest at G1 phase.

We further assessed the activity of AK-I-190 in apoptosis induction (Figure 4C,D). Flow cytometric analysis showed that AK-I-190 induced apoptosis to a greater extent than etoposide. In addition, Western blotting confirmed alterations in apoptosis markers. The pro-apoptotic proteins cleaved PARP and bax were increased, whereas the anti-apoptotic protein bcl-2 was decreased in a concentration-dependent manner. These results indicated that AK-I-190 induced G1 arrest and apoptosis in a time- and concentration-dependent manner.

### 2.4. AK-I-190 in Combination with Paclitaxel Inhibits Cellular Proliferation of a CRPC Cell Line

**Figure 5:**
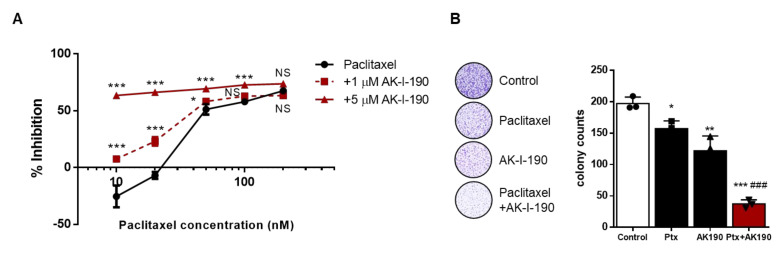
Enhanced anti-proliferative activity of AK-I-190 in combination with paclitaxel. (**A**) DU145 cells were treated with paclitaxel alone or in combination with AK-I-190 for 72 h and cell proliferation was evaluated by WST assay. (**B**) Clonogenic assay was performed with DU145 cells incubated with paclitaxel (1 nM) and/or AK-I-190 (5 μM) for 10 days. Cells were stained and viable colonies were counted. All determinations were made in 3 replicates in 3 different experiments and the values are mean  ±  S.D. * *p*  <  0.05, ** *p* < 0.01, and *** *p* < 0.001 vs. control; ^###^ *p*  <  0.001 vs. the paclitaxel-treated group.

To evaluate the effect of topoisomerase inhibition of AK-I-190 and its clinical relevance in CRPC treatment, we assessed the anti-proliferative activity of AK-I-190 combined with paclitaxel treatment in the DU145 cell line. Paclitaxel is a taxane that is used for chemotherapy of CRPC patients [16,17]. We first examined the anticancer activity of AK-I-190 combined with paclitaxel. The cellular viability of DU145 cells was remarkably decreased by the combination of AK-I-190 with paclitaxel compared to paclitaxel alone (Figure 5A). The anti-proliferative effect of AK-I-190 was also evaluated by colony forming ability assays (Figure 5B). Co-treatment of AK-I-190 with paclitaxel dramatically inhibited colony formation of DU145 cells compared with paclitaxel alone. Together, these results suggest that the topoisomerase II inhibitor AK-I-190 co-administered with paclitaxel effectively inhibits CRPC cell growth.

## 3. Discussion

In this study, we clarified molecular mechanism of AK-I-190 as a DNA intercalating catalytic inhibitor of topoisomerase II. We first assessed whether AK-I-190 is a poison or catalytic inhibitor of topoisomerase II. AK-I-190 did not generate the linear form of DNA in cleavage complex assay and sustained DNA-unbound topoisomerase II in band depletion assay. These results revealed that AK-I-190 is a catalytic inhibitor of topoisomerase II rather than a poison. Interestingly, co-treatment of AK-I-190 with etoposide reduced linear DNA formation compared to samples treated with etoposide alone in the cleavage complex assay. This demonstrates that AK-I-190 can be beneficially used in combination with etoposide for reducing genotoxicity while maintaining the efficacy of inhibiting topoisomerase activity. As a DNA intercalating catalytic inhibitor of topoisomerase II, AK-I-190 showed potent anticancer activity through G1 arrest and apoptosis induction. Moreover, AK-I-190 enhanced anti-proliferative activity of paclitaxel, a clinically using chemotherapeutic agent in CRPC.

AR independence in PCa is an important issue that determines the effectiveness of PCa treatment. At the beginning of disease, PCa is manageable, as it is sensitive to androgen-deprivation therapy. However, in a considerable proportion of PCa cases, disease progression with or without prostate serum antigen increase is observed after androgen-deprivation therapy [18]. These castration-resistant characteristics are closely correlated with metastasis and worse clinical outcome [4]. Therefore, developing better therapeutic options for CRPC treatment is critical.

Topoisomerase II is an enzyme that is essential for diverse cellular processes, such as transcription, replication, chromosomal condensation and segregation. As topoisomerase II is indispensable in proliferative cells, it has been regarded as an effective therapeutic target for the treatment of solid tumors for decades. Topoisomerase II inhibitors fall into two categories: poisons and catalytic inhibitors [19,20]. Topoisomerase II poisons act at the stage of DNA cleavage. This type of inhibitor effectively kills cancer cells by trapping the DNA-enzyme complex and blocking DNA re-ligation and enzyme release [20]. Most of the clinically used topoisomerase II inhibitors are poisons. Despite their highly potent anticancer activity, the use of these compounds has limitations because of their mechanism of action. Topoisomerase poisons stabilize the DNA-enzyme complex, which leads to DNA toxicity. Previous studies have reported that the genotoxicity induced by topoisomerase II poisons causes serious side effects, such as secondary malignancy [21].

A role of topoisomerase II in PCa has been reported. Amplification of the *TOP2A* gene, which encodes topoisomerase II, was shown to correlate with Gleason score and hormone resistance in PCa [22,23]. Multivariate analysis of PCa cases with systemic progression or death within 5 years after radical retropublic prostatectomy suggested several predictive factors including *TOP2A* amplification [24]. These previous studies indicated that topoisomerase II is not only a predictive factor, but also a promising therapeutic target in PCa. A topoisomerase IIβ–cleavage complex stabilized by topoisomerase II poisons was reported as a possible cause of oncogenic gene fusions, such as *TMPRSS2-ERG* [25,26,27]. *TMPRSS2-ERG* is one of the most common genomic alterations in PCa and is detected in up to 60% of PCa cases [24,28]. Considering that topoisomerase II inhibitors hardly possess isoform-selectivity between topoisomerase IIα and topoisomerase IIβ, topoisomerase II inhibition without the generation of unwanted DNA double-strand breaks is a critical factor in PCa treatment. 

To overcome the drawbacks in the clinical use of topoisomerase II poisons, numerous studies have been conducted to develop catalytic inhibitors with potent activities. Topoisomerase II catalytic inhibitors act at the other steps of the topoisomerase II catalytic cycle, except for the step of DNA-enzyme complex stabilization [13,29,30,31]. The most important side effect of a topoisomerase II poison is the DNA truncation that is induced by its mechanism of action. We confirmed that AK-I-190 did not induce DNA fragmentation in short-term treatment using the alkaline comet assay. Although DNA fragmentation is a marker of cellular death, it also represents generation of poison-induced DNA truncation under short-term treating condition. We then evaluated the DNA interaction with AK-I-190 by titrating AK-I-190 into ct-DNA, which resulted in induction of hyperchromicity in DNA absorbance spectra. In addition, AK-I-190 replaced EtBr, a DNA intercalating agent, indicating that AK-I-190 interacted with DNA in an intercalating manner. Molecular docking studies showed that AK-I-190 fits into DNA between base pairs and generates additional hydrogen bonds with phosphate groups in the two different chains of DNA. The anticancer activity of AK-I-190 in PCa was also confirmed. AK-I-190 induced G1 arrest and stimulated apoptosis in a dose- and time-dependent manner. Moreover, AK-I-190 combined with paclitaxel, a clinically used anticancer agent, inhibited CRPC cancer cell growth to a greater extent than the single treatments.

The targeting of additional or alternative therapeutic molecular targets can be a solution to improve treatment of patients in CRPC treatment. While topoisomerase II inhibitors have been used for PCa patients, there have been limitations in their clinical use because of their mechanism in stabilizing the DNA-enzyme cleavage complex, producing undesirable truncated DNA. Our results suggest that AK-I-190 may suppress AR-independent PCa cell growth by its potent inhibitory activity against topoisomerase II without genotoxicity. Though the results of this study suggest topoisomerase II inhibition as a realistic alternative for CRPC treatment, the molecular mechanism of how topoisomerase II inhibition affects AR-independent growth of CRPC cells has not been defined. In addition to our results, further studies are needed to elucidate the molecular relationship between topoisomerase II and CRPC.

## 4. Materials and Methods

### 4.1. Cell Culture and Cell Viability Assay

DU145 (human PCa cell line) cells were purchased from Korea Cell Line Bank (KCLB, Seoul, Korea) and cultured in RPMI 1640 (Welgene Inc., Gyeongsan, Korea) medium supplemented with 10% fetal bovine serum (FBS; Corning Inc., Corning, NY, USA) and 1% penicillin-streptomycin (HyClone Lab Inc., Logan, UT, USA) in a 37 °C, 5% CO_2_ incubator. All media were changed every 2 days and cells were sub-cultured as required. 

For WST assays, cells were seeded at 10,000 cells per well in 96-well cell culture plates on day 1. After 24 h, the cells were treated with AK-I-190 alone or in combination with paclitaxel in serum-free medium for 72 h. On day 5, Ez-cytoX (DoGen, Seoul, Korea) was added to each well and the cell viabilities were assessed according to the manufacturer’s protocol using Tecan Infinite M200 PRO Multi-Detection Microplate Reader (Tecan Group Ltd., Männedorf, Switzerland). 

### 4.2. Western Blot Analysis

Compound-treated DU145 cells were lysed with RIPA lysis buffer (Cell Signaling Technology, Danvers, MA, USA) containing 1% protease inhibitor cocktail (GenDEPOT, Katy, TX, USA). The lysed cells were incubated on ice for 20 min and centrifuged at 4 °C for 20 min at 12,000 rpm to obtain the supernatant. The protein was quantitated using a Pierce^TM^ BCA protein assay kit (Thermo Scientific, Waltham, MA, USA). Samples were mixed with 2× loading buffer (1 mM Tris-HCl, pH 6.8, glycerol, 10% SDS, bromophenol blue, beta-mercaptoethanol) and heated at 98 °C for 3 min. Samples were separated by SDS-PAGE and transferred to a PVDF membrane, and the membrane was incubated overnight with primary antibodies at 4 °C. Anti-topoisomerase IIα (#12286), anti-Cyclin D1 (#2922), anti-cleaved-PARP (#9541), anti-Bax (#2772), anti-Bcl-2 (#2872), anti-β-actin (#4967) and α-Tubulin (#2144) were from Cell Signaling Technology (Danvers, MA, USA). Anti-p27^kip1^ (#E11-0965B) was from EnoGene Biotech Co, Ltd. (NY, USA). The images were scanned by LAS-3000 (Fuji Photo Film Co., Ltd., Tokyo, Japan) and analyzed using Multi-Gauge Software (Fuji Photo Film Co., Ltd., Tokyo, Japan).

### 4.3. In Vitro DNA Topoisomerase II Relaxation Assay

Supercoiled pBR322 plasmid DNA (Thermo Scientific, Waltham, MA, USA) and recombinant human DNA topoisomerase IIα enzyme (Inspiralis Ltd., Norwich, UK) were incubated with DMSO or compound in assay buffer. The assay was performed according to the manufacturer’s protocol and a previous report [32]. The amount of supercoiled and relaxed DNA was quantified using an AlphaImager^TM^ (Alpha Innotech Corp., San Jose, CA, USA).

### 4.4. kDNA Decatenation Assay

Topoisomerase II kDNA decatenation assay was performed following the published method [33]. Briefly, the assay was performed using kDNA (TopoGen, Inc., Buena Vista, CO, USA) and reaction buffer according to the manufacturer’s protocol. Proteinase K (1.38 mg/mL, #P4850, Sigma Aldrich, St. Louis, MO, USA) was added to samples, and the samples were incubated at 55 °C for 30 min. The samples were loaded onto a 1.2% agarose gel (containing 0.5 μg/mL EtBr) and electrophoresed for 7 h at 30 V. DNA bands of each sample were visualized using an AlphaImager^TM^ (Alpha Innotech Corp., San Jose, CA, USA).

### 4.5. Topoisomerase II Cleavage Complex Assay

Cleavage complex assay was performed as described previously and reported [32]. Briefly, pBR322 (Thermo Scientific, Waltham, MA, USA) was incubated with topoisomerase IIα enzyme (Inspiralis Ltd.) and the reaction was stopped by adding stop buffer. The samples were digested with proteinase K (1.38 mg/mL, #P4850, Sigma, St. Louis, MO, USA) at 45 °C for 30 min. The samples were electrophoresed on an EtBr-containing agarose gel (1.5% agarose in 1X TAE buffer, 0.5 μg/mL EtBr). The linear DNA bands were visualized following the same method described as for the DNA topoisomerase II relaxation assay.

### 4.6. Band Depletion Assay

Band depletion assay was evaluated with etoposide and AK-I-190 according to the previously reported method [33]. Briefly, DU145 cells were treated with etoposide or AK-I-190 in serum-free medium for 2 h and harvested by trypsin. The harvested cells were washed with 1× PBS and incubated with the test compounds again at the same concentration for 15 min on ice. The cell pellets were lysed with a sonifier (Branson, MO, USA) in denaturing buffer at 20% amplification for 3 s and centrifuged at 12,000 rpm for 20 min. The supernatant was subjected to Western blotting on an 8% SDS-PAGE gel.

### 4.7. Alkaline Comet Assay

Alkaline comet assay was performed as described previously [12]. The assay was performed using single-cell gel electrophoresis with a Comet Assay Kit (Trevigen Inc., Gaithersburg, MD, USA) according to the manufacturer’s protocol. DU145 cells were treated with etoposide and AK-I-190 to evaluate DNA toxicity. DNA comet tail images were captured using an Axio Observer Inverted Microscope (Carl Zeiss Co. Ltd., Jena, Germany). Spots of DU145 cells were randomly selected for imaging, and the DNA tail length was calculated using image analysis software (Komet^TM^ 5.5, Andor Technology, Belfast, UK).

### 4.8. Potassium Iodide (KI) Quenching Assay

KI quenching assay was performed according to the previously reported method [34]. Fluorescence intensity of AK-I-190 was taken with or without ctDNA (#D1501, Sigma Aldrich, St. Louis, MO, USA) and with KI. Emission was recorded at 380 nm (excitation at 320 nm).

### 4.9. Competitive EtBr Displacement Assay

Competitive EtBr displacement assay was conducted according to the previously reported method [32]. The fluorescence intensity of EtBr-ctDNA (Sigma, St. Louis, MO, USA) was measured using a Tecan Infinite M200 PRO Multi-Detection Microplate Reader (Tecan Group Ltd.) with an emission spectrum of 520–700 nm (excitation at 471 nm).

### 4.10. In Silico Docking Study

To perform the docking study, the 3D structure for DNA (PDB code, 1Z3F) was prepared using the Flare tool provided by Cresset^TM^ Software (Cresset, Cambridgeshire, UK). The docking analysis between AK-I-190 and the DNA structure was performed using an energy grid defined by the ligand in the PDB structure, ellipticine. The three-dimensional structure file for AK-I-190 was also generated, minimized, and optimized before the docking process. Molecular docking simulations were conducted using an algorithm provided by Flare software packaged from Cresset.

### 4.11. Cell Cycle Analysis

DU145 cells were seeded into a 60 mm cell culture dish. After cells reached 70% confluence, the cells were treated with AK-I-190 at various concentrations or for various times and then harvested with trypsin. The harvested cells were centrifuged at 3200 rpm for 3 min and the supernatant was removed. Ethanol was added to the cell pellet and the sample was incubated overnight at −20 °C for cell fixation. The fixed cells were centrifuged at 3200 rpm for 3 min, mixed with PI staining solution (PI 1 µg/mL, RNase (#4642, Sigma Aldrich, St. Louis, MO, USA) 20 µg/mL, Triton X-100 0.05% in PBS) and incubated at 37 °C for 25 min. The stained cells were measured using a Fluorescence Activated Cell Sorting (FACS)-Caliber flow cytometer (BD Biosciences, San Diego, CA, USA).

### 4.12. Apoptosis Assay with Annexin V and PI Double Staining

After DU145 cells reached 70% confluence, the cells were treated with compounds for 36 h. All the suspended and adherent cells were harvested using trypsin centrifuged at 3200 rpm for 3 min. The cell pellet was resuspended in Annexin V binding buffer (#556547, BD Biosciences, San Diego, CA, USA). Annexin V-FITC (#556420. BD Biosciences, San Diego, CA, USA) and Propidium Iodide staining solution (#556463. BD Biosciences, San Diego, CA, USA) were added to the cell suspension and the sample was incubated for 20 min at room temperature. The stained cells were examined using a FACS-Calibur flow cytometer (BD Biosciences, San Diego, CA, USA) and data were analyzed using the BD Cell Quest Pro software.

### 4.13. Clonogenic Assay

DU145 cells (5 × 10^3^ cells/well) were seeded into six-well cell culture plates. The day after seeding, cells were treated with different concentrations of designated compounds for 10 days. Cells were then fixed and stained in a solution of 2% crystal violet in methanol. After washing cells with tap water, the colonies were photographed and counted using ImageJ software. Three different experiments were performed. 

### 4.14. Statistical Analysis

Statistics were assessed by one-way analysis of variance (ANOVA) or Student’s *t*-test with Prism V6.01 (GraphPad Software, San Diego, CA, USA). Differences between two values were statistically significant when the *p* value was <0.05. Data are presented as mean ± SD or mean ± SEM.

## Supplementary Materials

The following are available online at https://www.mdpi.com/article/10.3390/ijms222011246/s1.
