# Supplementary material for: AK-I-190, a New Catalytic Inhibitor of Topoisomerase II with Anti-Proliferative and Pro-Apoptotic Activity on Androgen-Negative Prostate Cancer Cells
Source: Int J Mol Sci. 2021 Oct 18;22(20):11246. doi: 10.3390/ijms222011246 (PMC8538266; doi:10.3390/ijms222011246)
Supplement: Supplementary file 1 [file ijms-22-11246-s001.zip › ijms-1412368-supplementary.pdf]

Supplementary Materials

AK-I-190, a New Catalytic Inhibitor of Topoisomerase II with Anti-Proliferative and Pro-Apoptotic Activity on Androgen-negative Prostate Cancer Cells

Kyung-Hwa Jeon [1,†], *Seojeong Park* [1,†], *Hae Jin Jang* [1], *Soo-Yeon Hwang* [1], *Aarajana Shrestha* [2], *Eung-Seok Lee* [2], *and Youngjoo Kwon* [1,*]

[1] College of Pharmacy, Graduate School of Pharmaceutical Sciences, Ewha Womans University, Seoul 03760, Korea; kjeon@ewha.ac.kr (K.-H.J.); tyfpzk@naver.com (S.P.); kate0915@hanmail.net (H.J.J.); hilarious06@naver.com (S.-Y.H.)

[2] College of Pharmacy, Yeungnam University, Gyeongsan 38541, Korea; aarajana90@gmail.com (A.S.)

[*]Correspondence: ykwon@ewha.ac.kr (Y.K.)

[†]Authors are equal contributors.

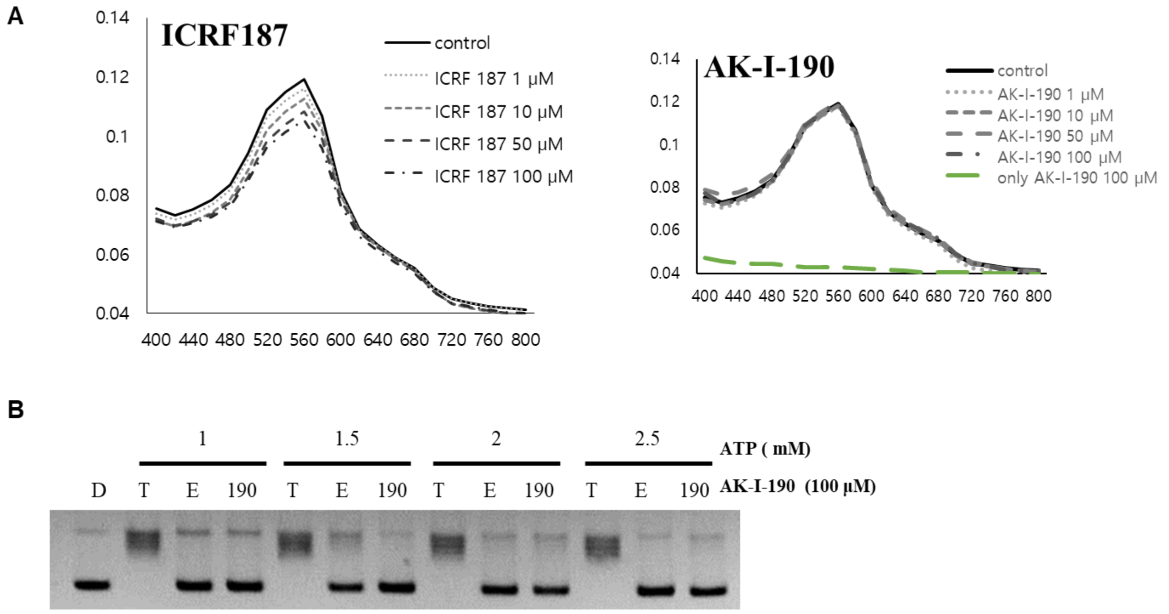

Supplementary Figure S1. Evaluation of mechanism of AK-I-190. (**A**) Metal chelating ability of AK-I-190 was assessed using Eriochrome Black T (EBT). AK-I-190 did not change the absorbance, whereas ICRF187, a metal chelating topoisomerase II inhibitor, reduced absorbance of Mg^{2+}-EBT complex by chelating Mg^{2+} ion. (**B**) ATP dependency in AK-I-190 mediated topoisomerase inhibition was evaluated by varying ATP concentration.
